# Prevention and Risk Assessment of Cardiac Device Infections in Clinical Practice

**DOI:** 10.3390/jcm13092707

**Published:** 2024-05-04

**Authors:** Andrea Matteucci, Carlo Pignalberi, Claudio Pandozi, Barbara Magris, Antonella Meo, Maurizio Russo, Marco Galeazzi, Giammarco Schiaffini, Stefano Aquilani, Stefania Angela Di Fusco, Furio Colivicchi

**Affiliations:** 1 Clinical and Rehabilitation Cardiology Division, San Filippo Neri Hospital, 00135 Rome, Italy; 2 Department of Experimental Medicine, Tor Vergata University, 00133 Rome, Italy

**Keywords:** CIEDs infection, prevention, health care cost optimization

## Abstract

The implantation of cardiac electronic devices (CIEDs), including pacemakers and defibrillators, has become increasingly prevalent in recent years and has been accompanied by a significant rise in cardiac device infections (CDIs), which pose a substantial clinical and economic burden. CDIs are associated with hospitalizations and prolonged antibiotic therapy and often necessitate device removal, leading to increased morbidity, mortality, and healthcare costs worldwide. Approximately 1–2% of CIED implants are associated with infections, making this a critical issue to address. In this contemporary review, we discuss the burden of CDIs with their risk factors, healthcare costs, prevention strategies, and clinical management.

## 1. Cardiac Device Infection and Risk Factors

Aging populations and advancements in medicine are driving a continuous rise in cardiac implantable electronic devices (CIEDs) implantation. While these devices demonstrably improve patient outcomes, they also introduce the potential for complications. Among these, infections represent a major concern, often leading to increased mortality, morbidity, and significant financial strain on healthcare systems [1,2]. However, accurately determining the precise rate of CIED infection proves challenging due to the inconsistent definitions used, the diversity of patient populations studied, and the varying methodologies employed in both retrospective and prospective research. The risk of developing cardiac device infections (CDIs) changes considerably based on the device implanted, the procedure adopted, and individual patient characteristics. [3]. CDIs can occur directly, during implantation or later procedures, affecting the leads, the generator, or both [4] or through bacteria traveling the bloodstream with a secondary infection of the device [5]. Gram-positive bacteria are the most commonly isolated bacteria in CDIs, in particular, staphylococci, which can cause bacteremia and pocket infections [6,7,8]. Other bacteria and fungi, however, have a very low incidence [6].

**Figure 1:**
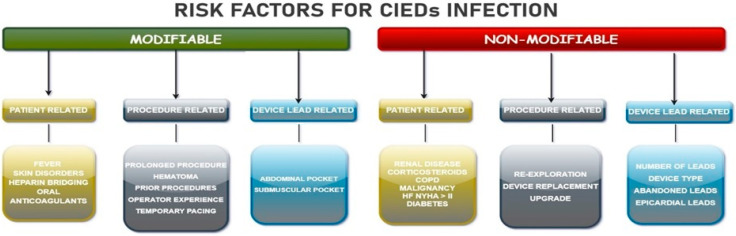
Classification of CIEDs risk factors. CIEDs = cardiac implantable electronic devices; COPD = chronic obstructive pulmonary disease; HF = heart failure; NYHA = New York Heart Association.

Categorically, various modifiable or non-modifiable factors can heighten the risk of CDIs: those related to the patient, the procedure, and the device itself [9] (Figure 1). It should be emphasized that different risk factors contribute differently to the studies conducted. Most are consistently recognized as being responsible for an increase in CDIs, such as pocket hematomas [10], re-exploration for lead reposition, and procedure time [11]. 

Certain patient characteristics have also been identified as potential risk factors for CDIs, for example, advanced age, female gender, and comorbidities such as diabetes, hypertension, and chronic kidney disease [9]. Also, the type of device itself can influence the risk of CDIs. The complexity of stratifying and preventing the various host- and procedure-related factors brought to the development of risk scores to stratify patients in low and high risk based on their susceptibility. The literature on infections of cardiac devices is predominantly retrospective [12,13], with a single prospective trial [14] being the exception. As of present, guidelines continue to approach study findings with caution, prioritizing recommendations that have undergone rigorous testing in the literature and have been validated through clinical trials. However, these studies can still offer valuable insights into the most vulnerable populations, aiding in the optimization of available resources and the mitigation of CDI risk.

## 2. Prevention Must Come First

**Figure 2:**
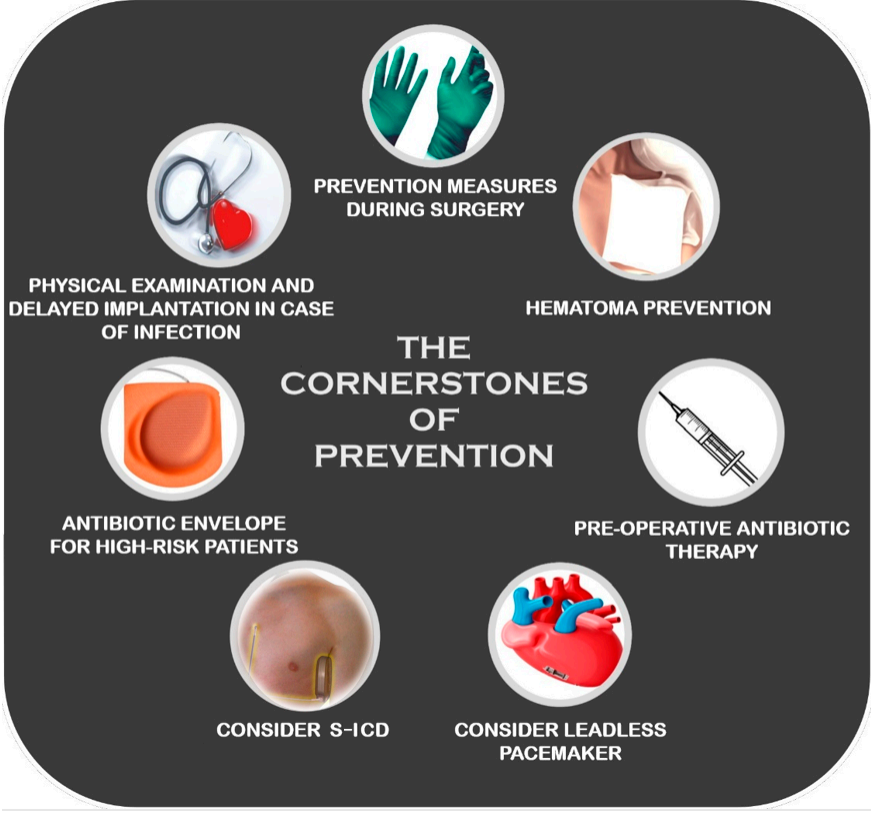
Main prophylactic tools to be applied at all times to prevent CDIs.

Prioritizing prevention is key to the optimal management of CDIs. Each patient demands a meticulous assessment of implant risks versus potential benefits. When infection risk is high, delaying implantation for observation or adopting long-term antibiotic therapy should be considered. Implantation should be postponed until afebrile status is sustained for at least 24 h in patients exhibiting fever or infection signs [15]. In high-risk individuals requiring defibrillation but not pacing, subcutaneous implantable cardioverter-defibrillators (S-ICDs) represent the best option. When pacing is required, epicardial systems offer an advantage over transvenous approaches [16] as well as leadless pacemakers, touted for lower infection susceptibility [17,18]. Anticoagulation therapy remains a topic of constant debate. Current recommendations suggest discontinuing therapy in patients at low thromboembolic risk and continuing anticoagulation in patients at higher risk of embolic events or mechanical valves. The recommendations extend to all types of anticoagulants [19]. Clearly, the concurrent use of anticoagulants and antiplatelet drugs multiplies the risk of bleeding. Therefore, it is prudent to consider the discontinuation of antiaggregant therapy in patients with an elevated risk of bleeding [20]. This strategy may mitigate complications such as pocket hematoma, which has historically been associated with an increased risk of CDIs. Antibiotic prophylaxis plays a crucial role in minimizing the risk of CIED infection, constituting a cornerstone of current clinical practice, achieving a relative risk reduction of up to 95% [21,22]. Timely administration is crucial, ideally within 60 min before the incision with a first-generation cephalosporin (cefazolin 1–2 g), to ensure sufficient tissue concentration [21]. For patients with cephalosporin allergies, vancomycin (15 mg/kg) represents the preferential alternative and should be initiated 90–120 min before the incision (Figure 2).

## 3. Available Tools and Appropriate Applications

Over time, various tools and materials were developed and tested to reduce infection risk in surgery. Among them, the TYRX [Medtronic, Minneapolis, MN, USA] is an antibacterial mesh that releases minocycline and rifampicin for 7 days to prevent infection and biofilm formation, fully dissolving in 9 weeks. Their effectiveness in reducing CDIs is well documented; however, the costs involved still limit their use to high-risk patients undergoing repositioning, repeated generator replacement, or other recognized high-risk factors for infection [23]. The importance of cost-effective utilization has caught the attention of many in recent years, and several ongoing registries and studies are testing the economic sustainability in different healthcare settings to generalize the use of this valid tool [24,25,26,27]. Recently, a study developed a score that can predict the risk of CIED infection by facilitating the allocation of cost-effective antimicrobial envelopes to high-risk patients [28]. Gentamicin-impregnated collagen sponges (GICSs) have been proposed for the same purpose [29,30]. This tool is used as a topical adjunct for perioperative antibiotic prophylaxis [31]. Gentamicin is an aminoglycoside that possesses a broad-spectrum bactericidal activity against frequent CIED pathogens like Staphylococcus spp. and aerobic Gram-negative bacteria and stands as a potential therapeutic option [32,33]. Notably, even as monotherapy, in vitro experiments have demonstrated its effectiveness against established staphylococcal biofilms [28,34]. However, systemic administration of gentamicin carries significant risks, including potential nephrotoxicity and ototoxicity. Localized delivery of gentamicin directly to the potential infection site could mitigate these concerns. Previous research has shown reduced infection rates when biological envelopes with different antibiotic solutions are used during CIED implantation [35]. Compared to TYRX, this tool has been studied rarely in CIED implantation [29]. However, there are several multicenter experiences and meta-analyses of the use of GICSs in various types of surgery. In cardiac surgery, they have been shown to reduce sternal wound infections in patients at high risk of infection [36]. Their efficacy has also been tested in colorectal surgery for the prevention of surgical wound infection. Although their costs are lower than other tools, the lack of specific studies on CIEDs implantation does not allow their routine use. Nowadays, other materials, support, or local antibiotic/antiseptic delivery systems lack sufficient evidence and require further study.

## 4. CIEDs Infection: Impact on the Healthcare System

CIED infection is well-defined by the recent European Heart Rhythm (EHRA) consensus paper [37] and the latest European Society of Cardiology (ESC) guideline for the management of endocarditis [38]. The new document also provides valuable insights by recommending antibiotic prophylaxis in high-risk patients for infective endocarditis (IE) undergoing high-risk dental procedures (dental extractions, oral surgeries, root canal treatments). Once dental implants are placed in high-risk patients, professional dental hygiene and follow-up should be conducted at least twice yearly under antibiotic coverage when indicated. The aim of prophylaxis is primarily to protect against oral streptococci [37]. Convincing evidence regarding the relationship between bacteremia from non-dental procedures and subsequent IE risk has not been presented. Antibiotic prophylaxis in high-risk patients undergoing non-dental medical procedures remains a subject for consideration. Regarding cardiac surgical interventions, it is strongly recommended to eliminate potential sources of dental sepsis at least 2 weeks before the implantation of a prosthetic valve or other intracardiac or intravascular foreign material unless the latter procedure is urgent. Specific antibiotic prophylaxis regimens exist for many specific devices (TAVR, CIEDs) [38]. The attention that preventive and prophylactic measures play on CIED infection tries to stem the increasing costs associated with their management and the impact on patient’s life expectancy. Different studies reported a 5–8% hospital mortality rate due to CIED infection [2,39]. This rate increases for individuals with substantial comorbidities such as heart failure or kidney issues, particularly when the infection involves the CIED endocarditis rather than the pocket [40]. Aside from death, complications following CIED infection within the hospital setting can include issues arising from lead extraction. These complications range from emergency thoracotomy for perforations and arteriovenous fistulas to septic pulmonary emboli, arrhythmias, and sepsis. Reimplanting a new device after removal might lead to uncommon yet possible complications such as recurrent infections [41]. Additionally, the presence of end-stage renal failure worsens the prognosis considerably. Long-term follow-up for individuals with prior CIED infection showed a high mortality risk [42]. The costs associated with such a wide variety of possible complications are extensive and documented well around the world. Further variability is also due to the type of healthcare system, as well as the varying incidence of CDIs themselves. 

In North America, an analysis of over five thousand Medicare patients showed that the average cost for each patient requiring CIEDs extraction and replacement was sixty-two thousand dollars. The average costs decrease for those who had the device extracted but not reimplanted, while they rise to over seventy-seven thousand dollars for patients hospitalized for CIED infection but not undergoing removal [43]. Hospitalization, which included the cost of device system extraction and replacement, was the main driver of cost. Non-extracted patients with infection-related hospitalization had the longest hospital stay and the largest use of resources. The significant financial burden associated with CIED infection highlighted in North America is confirmed by analyses performed in Europe in different countries [44,45]. The direct costs of infections are related to the duration of hospital stay, diagnostic testing, antibiotic treatment, extraction procedure, CIEDs reimplantation procedural, and device costs. An interesting analysis reveals that two categories of direct costs account for more than two-thirds of the total costs of treating CIED infection [46]. First of all, hospital stay costs (39.1% of total costs) and CIEDs, including leads (31.2% of total expenditure). The costs of the devices depend on the type of device used, with cardiac resynchronization therapy (CRT) being the most expensive device on average. The distribution of expenditures for patient treatment depended on the type of device. For pacemaker and implantable cardioverter defibrillator (ICD) systems, the largest cost category is hospital stay. Among patients with the relatively most complex CRT system, the device costs are the highest when compared with other categories. The cost of devices increased proportionally to their complexity, while the cost of hospitalization depended on the length of hospital stay. Interestingly, the longest stays were observed in patients with the most complex systems and was due to the need to treat infection before reimplantation. Considering the aforementioned costs of hospitalization, the costs of devices used for reimplantation, and other financial outlays, in many cases, the treatment of CIED infection complications was a source of financial loss for the hospital. This was confirmed by different studies in which the costs per patient with a new infection with or without replacement procedures occurring within 1–2 years after CIED implantation were similar. For infections associated with new procedures, reimbursement averaged 63% to 71% of the average total cost of CIEDs with and without replacement procedures, respectively [1].

## 5. Clinical Presentation of CIEDs Infection

Two key manifestations are identified in the clinical presentation of CIED infection: device pocket infections and lead-related endocarditis. While the definitive therapeutic approach for both these clinical manifestations is hardware removal, it is important to emphasize that pocket infection should be distinguished from superficial wound inflammation, as the latter typically responds to medical therapy alone. The device pocket represents the most frequent site of infection, affecting nearly two-thirds of all infections, whereas lead involvement is associated with higher rates of complications and mortality [47]. It is commonly accepted that infections occurring within one year of implantation are likely related to contamination during the procedure and predominantly involve the pocket with potential spread to the catheters, while those manifesting later seem to be caused by bloodborne pathogens primarily affecting the leads. However, the retrograde progression of infection from the bloodstream to the pocket has been described [48]. In the latter case, the entry points for pathogens are generally cutaneous, oral, lower respiratory tract, gastrointestinal, or urinary tract infections [48]. Uncomplicated pocket infections are defined as being confined to the device location without systemic signs and symptoms, typically presenting with erythema, warmth, abscesses, adhesions, and erosions. If the generator or proximal leads are exposed, the device should be considered infected regardless of microbiological results [8]. Nevertheless, pocket infections should never be regarded as localized CIED infections as they are often complicated by microbial spread to the leads and/or bloodstream through contiguity. Early stages of infection may be difficult to distinguish from post-implantation wound inflammation, often resolving within a month of implantation. In these circumstances, percutaneous diagnostic puncture should always be avoided as it only increases the risk of further contamination [49]. Complicated pocket infections are defined by evidence of lead or endocardial involvement, positive blood cultures, and the presence of systemic signs and symptoms of infection. CIEDs-related endocarditis (CIEDs/IE) is defined by bloodstream infection with or without evident lead vegetations or valvular apparatus involvement. As mentioned earlier, it may result from bloodstream dissemination of pocket infection or secondary lead involvement due to bacteremia from other sources. Symptoms may be nonspecific such as chills, night sweats, and fever responding positively to antibiotic therapy and recurring upon its cessation, although fever and systemic manifestations may often be absent. In the presence of lower respiratory tract infections or rheumatologic symptoms (spondylodiscitis or osteomyelitis), CIED infection should always be suspected. 

The diagnosis of CIEDs/IE still relies on modified Duke criteria with the incorporation of new imaging techniques such as (18)F-fluorodeoxyglucose (FDG) positron emission tomography (PET), single-photon emission computed tomography (SPECT)/CT and radiolabeled white blood cells scintigraphy in the EHRA international CIEDs infection criteria [37], improving sensitivity and defining the following diagnostic classes:

- Definite CIEDs clinical pocket/generator infection (swelling, erythema, warmth, pain, purulent discharge/sinus, pocket deformation, erosion, exposure)
- Definite CIEDs/IE (2 major criteria or 1 major + 3 minor)
- Possible CIEDs/IE (1 major criterion + 1 minor or 3 minor)
- Rejected CIEDs/IE without the aforementioned criteria for IE.

As previously mentioned, Coagulase-negative staphylococci represent the most frequently encountered infectious agents in CIEDs infection (42–77%), followed by *Staphylococcus aureus* (10–30%), *Gram-negative bacilli* (6–11%), *Streptococcus* spp. (3–10%), *Enterococcus* spp. (0.4–10%), *Cutibacterium* spp. (0.8–8%), and *fungi* (0.4–1.4%), predominantly *Candida* spp. [8]. Methicillin-resistant staphylococci (both coagulase-negative and positive) alone account for approximately one-third of all cases [37]. *Staphylococcus aureus* is the leading cause of bacteremia and early CIEDs infection, and along with Gram-negatives, represents the microorganisms responsible for clinically severe infections more frequently associated with lead-related endocarditis. Several clinical studies have endeavored to identify predictive factors for CIEDs/IE in patients with *Staphylococcus aureus* bacteremia in the absence of pocket infection signs, such as recurrent bacteremia after an appropriate period of antibiotic therapy, persistent bacteremia for more than 24 h, presence of an ICD, prosthetic heart valve, and bacteremia within 3 months of device implantation [50].

## 6. Microbiological Investigations and Diagnostic Imaging

The identification of the etiological agent in CIED infection is of paramount importance for initiating appropriate antibiotic therapy; thus, it is crucial to conduct microbiological cultures before therapy initiation. Three sets of blood cultures, spaced 30 min apart, should be obtained in CIED patients with fever but without clear signs of local infection or endocarditis before starting antibiotic therapy. In hemodynamically unstable patients, this may not be feasible, and empirical therapy should be initiated after obtaining two sets of blood cultures to be optimized based on the results. Each positive blood culture, including single positivity for coagulase-negative staphylococci or Gram-negative bacteria, warrants further diagnostic techniques to exclude CIEDs infection [e.g., transthoracic (TTE) and transesophageal (TEE) echocardiography, FDG PET/CT, WBC SPECT/CT)] as not all patients with CIEDs and positive blood cultures have underlying lead infections [51]. Positive blood cultures should be repeated at 48–72 h and every 48 h thereafter until negativity is achieved. In cases of negative blood cultures after 5 days with a strong clinical suspicion of CIED infection, prolonged incubation (10–14 days) is advisable, and molecular techniques such as DNA amplification with PCR and genetic sequencing may be considered for the diagnosis of atypical pathogens (e.g., mycobacteria and fungi) [8]. Additionally, some Gram-positive species may require longer incubation times (e.g., *Cutibacterium acnes*), potentially causing purulent pocket infections with negative endocarditis findings after 3 days. Furthermore, incidental findings of masses adhering to leads without clinical signs of infection, possibly thrombotic in nature, should be considered. In such cases, four sets of blood cultures and inflammatory markers over 2–4 days should be obtained. If all results are negative, clinical and echocardiographic follow-up is warranted, and anticoagulant therapy should be considered, although the neoplastic nature of the mass cannot be excluded [52]. Upon CIED extraction, tissue samples from the pocket should undergo culture and Gram staining as they have shown higher sensitivity for identifying involved microorganisms compared to swab cultures [53]. The entire extracted device and proximal and distal lead fragments should be sent to the microbiology laboratory for sonication to disrupt the biofilm and increase the likelihood of isolating microorganisms for culture [54]. However, lead tip cultures may be positive in patients with pocket infection due to contamination during the extraction procedure. In the absence of other evidence of lead-involved endocarditis (blood cultures, echocardiography, nuclear imaging), a positive catheter tip culture does not warrant prolonged antibiotic therapy. The evaluation of a patient with suspected CIED infection (positive blood cultures, suspicious symptoms, evidence of septic pulmonary emboli) should always include both transthoracic TTE and TEE. TTE allows for better assessment of pericardial effusion, left ventricular function, and pulmonary pressures, while TEE enables better visualization and measurement of vegetations adherent to catheters or valve apparatuses. Intracardiac echocardiography (ICE) exhibits high sensitivity for detecting vegetation, and thus, vegetation detected via ICE can be considered a major diagnostic criterion. It may be considered in cases of negative TTE and TEE results, although largely not utilized for diagnostic purposes but for monitoring during the extraction procedure. The advantages of these techniques include the potential for early diagnosis, the ability to resolve ambiguous situations of possible CIED infection (e.g., differential diagnosis between superficial wound infection and pocket infection or evaluation of cases with vegetation findings in asymptomatic patients with negative blood cultures, or patients with systemic symptoms without further diagnostic findings) and obtaining additional information regarding extracardiac infection sites. (18-FDG)-PET/CT exploits the accumulation of 18-FDG in metabolically active tissues and should be optimized through dietary preparation to reduce physiological 18-FDG uptake by cardiomyocytes (low-carbohydrate meal followed by at least 4 h of fasting before the examination) [55]. This technique has shown sensitivity and specificity of 96% and 97%, respectively, in diagnosing pocket infections in a meta-analysis [56]. Lower values were observed for diagnosing endocarditis (76% and 83%, respectively). These characteristics play an important role in diagnosing doubtful pocket infections. Scintigraphy labeled WBC SPECT/CT has demonstrated lower sensitivity but higher specificity than (18-FDG)-PET/CT for CIEDs infection [57]. 

If available, (18-FDG)-PET/CT is the preferred nuclear medicine investigation due to its higher sensitivity, rapidity, and practicality [58]. The role of WBC SPECT/CT is limited to cases where the initial examination is unavailable or has provided results of non-univocal interpretation.

## 7. Device Explantation and Reimplantation 

The effective management of a defined CIED infection relies on the complete removal of all hardware components of the system, including the generator and all active and abandoned leads, both endocardial and epicardial, including fragments, and any further permanent vascular access [59]. This foundational treatment principle applies to both systemic infections and those limited to the pocket [60]. Additionally, timely treatment and rapid diagnosis are of paramount importance; indeed, system removal within three days of hospitalization leads to a significant reduction in in-hospital mortality. Complete device extraction should be performed when valve replacement or repair intervention is necessary due to endocarditis, even in the absence of CIEDs infection evidence, as CIEDs may serve as substrates for pathogen persistence [61]. Device removal is also indicated for patients with infective endocarditis without defined CIED involvement. In cases of occult bacteremia or fungemia, therapeutic strategy varies depending on the identified microorganism. A single positive test for Staphylococcus aureus or *Candida* spp. is sufficient to suggest system extraction. Conversely, Coagulase-negative Staphylococci and *Cutibacterium* spp. require high-grade bacteremia (two or more separate positive blood cultures) for a specific diagnosis. In these circumstances, procedural risks associated with CIED removal are significantly lower than the mortality or infection recurrence rates observed with alternative strategies such as antibiotic therapy or generator extraction with lead preservation [60]. For *Streptococcus* spp. and *Enterococcus* spp. bacteremia, extraction may be considered as first-line treatment or second-line in cases of persistent or recurrent bacteremia despite adequate antibiotic therapy. For Gram-negative bacteremia (excluding Serratia/Pseudomonas) or Pneumococci, medical therapy is the initial approach, with extraction upon persistent/recurrent bacteremia [8,62,63]. When indicated, transvenous lead removal is the most recommended technique, with low mortality and major complication rates [8]. Leads implanted for at least two years are technically more difficult to extract and should be performed by experienced operators. Different lead types pose various challenges during removal: ICD leads, with one or two coils leading to more extensive adhesions, are more prone to procedural complications, especially in the presence of a caval coil; the same applies to those with passive fixation compared to active fixation. 

Transvenous lead extraction (TLE) has evolved significantly, moving from single-force methods to a more nuanced approach balancing traction and countertraction. Modern TLE utilizes a combination of traction and countertraction exerted by a dissecting sheath. Traction, sometimes aided by a locking stylet, allows for controlled force transmission to the lead tip, minimizing breakage. Countertraction is applied to advance the sheath along this railroad, facilitating the dissection of the lead from surrounding tissue adhesions [64]. Early dissecting sheaths were made of various materials like polypropylene, Teflon, or steel, each with different properties. Teflon offered flexibility but limited dissection ability, while polypropylene provided rigidity for tougher adhesions but struggled with curves. Steel sheaths were used for particularly dense scar tissue at the entry point to the central vasculature. Two primary methods exist for delivering thermal energy to the sheath tip during lead extraction: laser and radiofrequency energy. These techniques demonstrate improved efficacy compared to traditional sheaths, potentially requiring less force for lead dissection. The first powered sheath introduced was the laser tool, adapted from percutaneous coronary intervention procedures in the mid-1990s. This technology utilizes pulsed ultraviolet light with a specific wavelength (around 308 nm) and high repetition rate (40–80 Hz) to ablate surrounding tissue [65]. The laser energy targets lipids and proteins at the cellular level, disrupting vital bonds and causing tissue disintegration. Electrosurgical dissecting sheaths instead utilize radiofrequency energy for lead extraction and have also shown effectiveness compared to conventional techniques. Despite their initial promise, radiofrequency sheaths have largely been replaced by rotational tools due to limited market share and eventual discontinuation [66]. In recent years, rotational tools have emerged as a valuable addition to the armamentarium for TLE. These devices employ a motorized mechanism featuring a stainless-steel dissecting tip that rotates upon user activation. Similar to laser and basic mechanical sheaths, the rotational tool is inserted over the lead and advanced with a combination of forward pressure and counter-traction. The tool disrupts fibrous tissue adhesions around the lead, facilitating its removal. Early rotational tools, monodirectional, faced limitations with lead wrapping during extraction. This led to the development of new devices featuring bidirectional rotation for improved efficacy [66]. The newer rotational sheaths exhibit design differences compared to the previous, particularly in terms of stiffness and tip configuration [67]. Their ability to effectively manage challenging cases, especially those with extensive scar tissue, makes them a valuable addition to the lead extraction toolkit.

In patients with large-sized vegetation adhering to catheters (over 20 mm), surgery should be considered due to the potential risk of pulmonary embolism with transvenous methods. In these cases, a thorough risk-benefit assessment is necessary as a surgical threshold has not yet been officially defined, and this approach is burdened with increased morbidity [38]. Percutaneous removal of large vegetations using aspiration techniques and veno-venous extracorporeal circulation filtering has shown beneficial effects in reducing post-operative sepsis and pulmonary embolization [68]. Management of the generator pocket requires particular attention, with complete debridement, excision of the capsule, and all non-absorbable sutures followed by abundant irrigation with saline [69]. In CIEDs infection with epicardial leads, complete lead removal is recommended in cases of defined involvement after adequate evaluation comparing surgical risk versus infection-related mortality risks [70]. In cases of localized pocket infection without defined involvement of distal portions of the conductors, these can be spared from removal, which will only involve the generator and proximal lead portions. In patients with superficial wound infections (in the absence of evident pocket involvement), system extraction can be avoided in favor of empiric antibiotic therapy lasting 7–10 days [38]. Following the removal of an infected device, the need for a new CIED implantation must be evaluated, which typically occurs in about one-third of cases. Implantation of the new device should be contralateral, preferably opting for device types with lower infection risk (leadless devices, subcutaneous ICD, epicardial leads). Reimplantation timelines vary depending on the scenario: patients with documented valve vegetations on TEE should be reimplanted at least 14 days after the last negative blood culture result. In cases of limited lead involvement or bacteremia without demonstration of vegetations, reimplantation may suffice 72 h after the last negative blood culture. For pocket-limited infections, new implantation can occur upon complete local healing. Although not considered standard practice, limited experience supports same-day CIED reimplantation following extraction [71]. For patients at high risk of CIEDs requirement, the best bridging options include active fixation lead placement via the internal jugular, connected to an external generator, or wearable defibrillator placement for non-pacing patients.

The choice of the optimal antibiotic therapy is closely dependent on patient characteristics and the type of identified infectious involvement; for further elaboration, refer to the previously cited works [37,38]. Recent updates from ESC guidelines categorize therapy into two phases: an initial critical phase and a continuation phase. The initial phase occurs during hospitalization, lasting up to 2 weeks, and involves the administration of intravenous antibiotics with rapid bactericidal activity. Device removal is planned during this phase. Following this period, clinically stable patients in a stable home environment may complete antibiotic therapy at home with oral or intravenous regimens for at least 6 weeks to eliminate resting bacteria and prevent potential recurrences. Another novelty concerns the duration of therapy: in CIEDs infections not caused by S. aureus and without valvular involvement or the presence of vegetations, if follow-up blood cultures are negative, consideration can be given to a treatment duration of only 2 weeks post-extraction. Conversely, extending treatment to 4–6 weeks post-extraction should be considered in the presence of septic emboli or prosthetic valves.

## 8. Discussion

In this paper, we highlight various factors in many important manuscripts that contribute to increasing CDIs categorized as patient-related (comorbidities, age), procedure-related (repositioning, pocket hematoma), and device-related (device complexity). Recognizing their role paves the way for risk stratification and tailored interventions. Accurate infection rate estimation remains elusive due to different definitions and study methodologies, but understanding risk factors and implementing preventive strategies are crucial. Prospective studies are still limited, but the existing literature offers valuable insights. Prevention is the cornerstone of management. Meticulous risk assessment is crucial, with potential benefits weighed against infection risks. Delaying implantation or opting for long-term antibiotics might be necessary for high-risk patients. S-ICDs and leadless pacemakers offer alternatives when feasible [72]. Anticoagulation management is challenging, with discontinuation considered for low thromboembolic risk and continuation for high-risk scenarios. Available tools to prevent CDIs like TYRX hold promise, but cost limitations restrict their use to high-risk scenarios as well as GICSs for which studies are lacking. Other preventive strategies for cardiac device infections include pocket instillations of antibiotic agents, the benefit of which is still debated [73,74]. An application of the PADIT score recently proposed could be a valid approach to minimize the financial costs driven by hospital stays, device replacement, and various interventions [75]. Finally, there is still a lack of interest in the remote management of patients with CDI, using the tools made available by CIEDs capable of performing multi-parametric evaluations and providing information on a wide variety of parameters, as has been demonstrated in last years due to pandemic conditions [76]. There are few experiences aimed at evaluating their application in CIED infection [77], but technological advancement will certainly provide time for more and more valid instruments capable of covering these needs as well.

## 9. Conclusions

CIED infection presents a complex challenge. Understanding risk factors, implementing proven preventive strategies as with antibiotic prophylaxis, and judicious use of prevention tools are crucial to ensure optimal resource allocation. By prioritizing prevention, investing in research, and adopting a multi-disciplinary approach, we can mitigate the burden of CIED infection and improve patient outcomes while safeguarding healthcare system sustainability.
